# How many landmarks are enough to characterize shape and size variation?

**DOI:** 10.1371/journal.pone.0198341

**Published:** 2018-06-04

**Authors:** Akinobu Watanabe

**Affiliations:** 1 Department of Anatomy, New York Institute of Technology, Old Westbury, New York, United States of America; 2 Division of Paleontology, American Museum of Natural History, New York, New York, United States of America; 3 Richard Gilder Graduate School, American Museum of Natural History, New York, New York, United States of America; 4 Life Sciences Department, Vertebrate Division, Natural History Museum, London, United Kingdom; Monash University, AUSTRALIA

## Abstract

Accurate characterization of morphological variation is crucial for generating reliable results and conclusions concerning changes and differences in form. Despite the prevalence of landmark-based geometric morphometric (GM) data in the scientific literature, a formal treatment of whether sampled landmarks adequately capture shape variation has remained elusive. Here, I introduce LaSEC (Landmark Sampling Evaluation Curve), a computational tool to assess the fidelity of morphological characterization by landmarks. This task is achieved by calculating how subsampled data converge to the pattern of shape variation in the full dataset as landmark sampling is increased incrementally. While the number of landmarks needed for adequate shape variation is dependent on individual datasets, LaSEC helps the user (1) identify under- and oversampling of landmarks; (2) assess robustness of morphological characterization; and (3) determine the number of landmarks that can be removed without compromising shape information. In practice, this knowledge could reduce time and cost associated with data collection, maintain statistical power in certain analyses, and enable the incorporation of incomplete, but important, specimens to the dataset. Results based on simulated shape data also reveal general properties of landmark data, including statistical consistency where sampling additional landmarks has the tendency to asymptotically improve the accuracy of morphological characterization. As landmark-based GM data become more widely adopted, LaSEC provides a systematic approach to evaluate and refine the collection of shape data––a goal paramount for accumulation and analysis of accurate morphological information.

## Introduction

Techniques for characterizing morphology provide a lens with which to describe, interpret, and analyze variations in form. In recent years, geometric morphometric (GM) methods have become widely adopted in morphological studies due to their efficacy in capturing, retaining, and visualizing shape information [1–4]. Typically, GM data comprise a set of two- or three-dimensional (2-D or 3-D) Cartesian coordinate points positioned on specimens and structures of interest. The raw coordinate data are then aligned to extract shape information and analyzed to investigate a wide range of topics in biology, engineering, and design.

**Fig 1:**
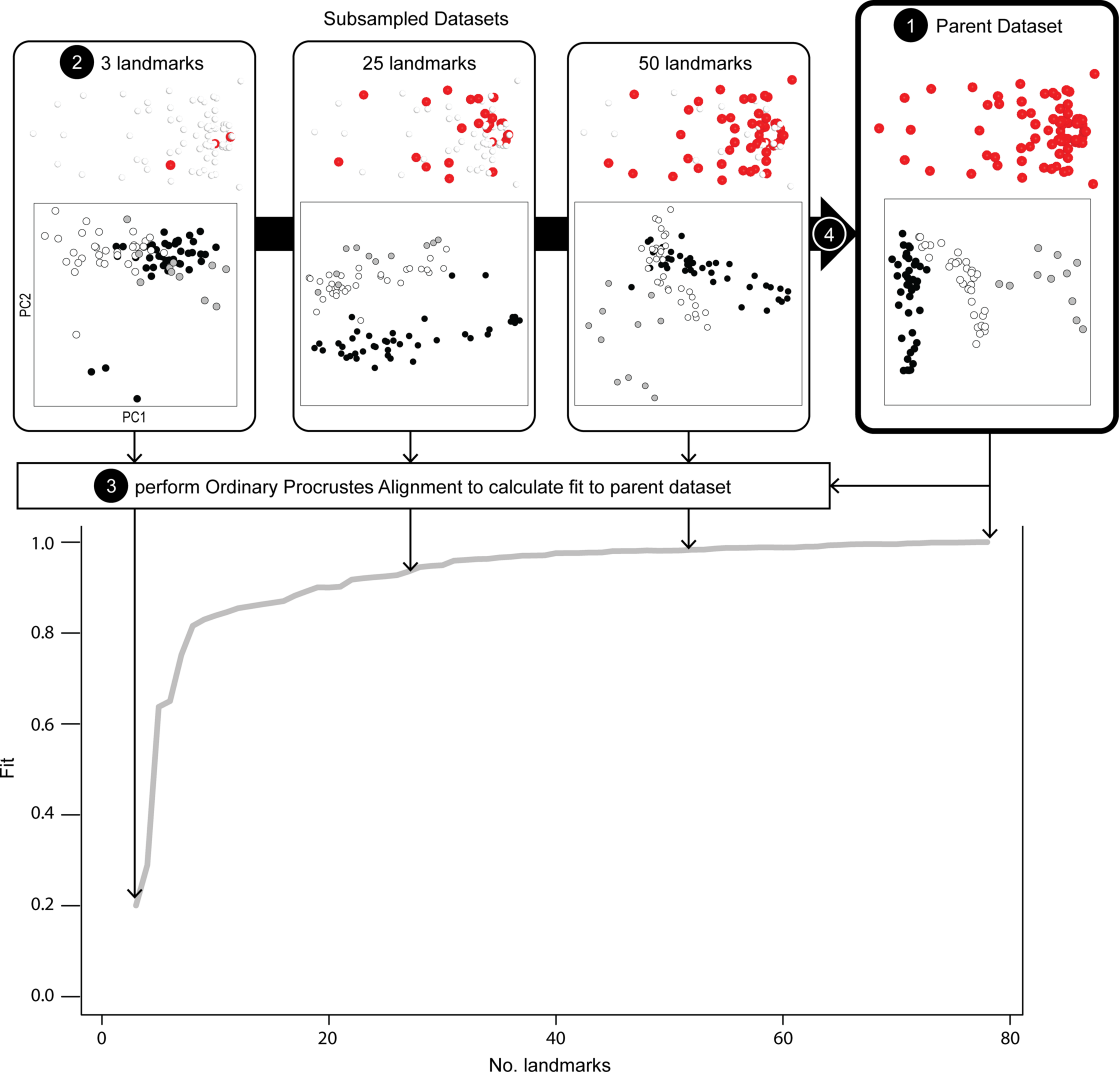
Schematic diagram of steps performed during a single iteration of LaSEC on a crocodylian skull dataset [25]. The procedure begins by extracting shape information from a coordinate dataset (“Parent dataset”), subsampling three randomly selected landmarks, then calculating its fit of specimen distribution to that of the parent dataset based on Procrustes sum of squares (PSS). Once PSS is recorded, one additional landmark is randomly selected and the fit value is calculated between the new subsampled data and the parent dataset. This process is repeated until all landmarks in the parent dataset are sampled. The adequacy of landmark sampling is assessed based on the extent of stationarity observed in the resulting curve. The morphospaces constructed from first two principal components (PC) of subsampled shape variation illustrate the drastic differences in the distribution of major crocodylian clades: Alligatoridae (black), Crocodylidae (white), Gavialidae (gray). Circled numbers correspond to in-text description of the procedure. Coordinate data visualized in Morpheus et al. version 1.8.0 [34] where sampled landmarks are red and unsampled landmarks are white.

As with any scientific data, the quality of GM data remains a crucial component of conducting sound research. Namely, do our data accurately reflect the shape variation of objects and structures under study? Using horse teeth, Cardini and colleagues examined the number of specimens needed for reliable estimation of mean shape [5]. Cardini also examined the congruence between datasets with both pairs of bilateral landmarks and those with only one side sampled, showing that the latter case exaggerates the variation along the midline [6,7]. A related issue in GM that has eluded systematic investigation is whether the landmark sampling is sufficient for characterizing morphological variation. This issue is critical because sampling too few landmarks will obscure local shape differences that drive global shape differences among specimens, ultimately generating spurious results biased by landmark choice (Fig 1: morphospaces). Poor landmark sampling is more probable in fields such as paleontology and archaeology, where the inclusion of important, but damaged or deformed, specimens prompt the removal of potential landmarks from analyses. However, sampling too many landmarks is also problematic due to increased work load required for data collection and analysis. Moreover, while oversampling landmarks may benefit visualization and estimation of shape [3,8]), the power of many standard statistical tests suffers as the number of shape variables exceeds the number of specimens [9,10]. Some digitization approaches, such as user-specified sampling of semi-landmarks along a curve (e.g., [11]) and automatic placement and alignment of landmarks on 3-D surfaces [12], provide the capacity to place hundreds to thousands of landmarks on specimens without first evaluating the need for such dense characterization of shape. As such, knowledge of whether a dataset adequately captures shape variation could drastically improve the practice of collecting landmark data overall.

Moreover, a theoretical notion important to landmark sampling is whether shape data are consistent. In GM the term “consistency” has typically referred to the precision in digitization (e.g., [13]) and in some cases, the congruence between morphometric data collected under different modes (e.g., [14]) or superimposition methods [9,15]. However, “consistency” in the context of sampling theory has been seldom studied in the GM literature, in which shape data converge to the true values as *n* → ∞, where *n* denotes the number of landmarks. Kent and Mardia examined the consistency in the estimation of mean shape by generalized Procrustes superimposition as more specimens are sampled, demonstrating that specimen sampling is statistically consistent with respect to mean shape, assuming isotropic errors at each landmark [16]. Yet, no study to date has studied the consistency in overall shape data with respect to landmark sampling. In addition, the statistical efficiency of landmark data also remains to be examined, where the variance around the estimated value decreases as *n* → ∞, where *n* signifies number of landmarks.

Here, I present a new R function called Landmark Sampling Evaluation Curve (LaSEC) to determine whether a landmark-based dataset has achieved stationarity in capturing shape, as well as size, information. I demonstrate its practical utility by performing LaSEC on published empirical data sets. In addition, I also investigate the theoretical issues of whether landmark data are statistically consistent and efficient using both simulated and empirical data. The output from LaSEC allows the user to evaluate the fidelity of landmark data and avoid sampling too few or too many landmarks. The function is included in the new LaMBDA (LandMark-Based Data Assessment) R package (www.github.com/akiopteryx/lambda; S1 Data).

## Materials and methods

### Analysis

The function LaSEC is coded in R [17] and utilizes the R packages geomorph [18] and vegan [19] for aligning coordinate data and calculating the degree of congruence between subsampled and full data sets, respectively. To run the function, the user specifies three items: (1) the coordinate data in the format of a 2-D matrix where specimens are rows and shape variables are columns, (2) the physical dimensionality of the data (i.e., 2-D or 3-D), and (3) the number of resampling rounds: lasec(coord.data, n.dim, iter). The coordinate data are assumed to be unaligned and complete, reflecting the size variation of the sampled specimens and without any missing data.

To measure the differences between the full (hereby “parent”) and subsampled datasets, the function uses the protest function in the vegan R package [19] to calculate the Procrustes Sum of Squares (PSS). This method [20] performs an ordinary Procrustes alignment [21] to superimpose the distribution of specimens in the subsampled data to that of the parent data. While the typical implementation of Procrustes alignment in GM studies involves translation, rotation, and scaling of coordinate data in physical 2-D or 3-D space, the alignment procedure here subjects the shape data to translation, rotation, and scaling in full, hyper-dimensional shape space. Described another way, the constellation of the specimens in the shape space of subsampled data are aligned to those in the shape space of the parent data. Because this alignment requires equivalent statistical dimensions in the two datasets, the function automatically adds columns of zeros to the subsampled data to match the dimensionality of the parent data (i.e., the same number of shape variables). The addition of zeros, or any single number, in this step does not alter the value of PSS because these dummy variables do not contribute any variation to the shape data. Hence, the distribution of specimens in the respective shape spaces remains the same with the addition of these dummy variables. To match the aesthetic of typical sampling rarefaction curves and Bayesian trace plots, the ‘fit’ between subsampled and parent data is measured as 1–PSS, such that values equal to one and near zero signify perfect and poor Procrustes fit, respectively.

Although the parent and subsampled datasets are in separate shape spaces, PSS compares the shape distribution of specimens in full statistical dimensionality, which is the basis of qualitative and quantitative shape analyses in GM studies. Other measures of congruence, such as correlation coefficients are limited if correlation is based on reduction of shape data into pairwise distances or few principal components of shape variation which may misrepresent congruence in full shape space ([22]). Correlation coefficients from two-block partial least squares analysis [23] uses the full dimensionality of datasets. However, it yields very high levels of correlation despite low qualitative and visual congruence between datasets (S1 Fig). This result is likely due to the subsampled dataset being a subset of the parent dataset, and thus, the method identifies linear combinations of data with strong correlations even with very low landmark sampling. For these reasons, PSS was utilized as a sensible metric for measuring the overall congruence between the subsampled and parent datasets.

Given this measure of fit, LaSEC conducts the following procedure (Fig 1: step numbers below correspond to circled numbers in figure):

1. As is typical for extracting shape data, perform a generalized Procrustes alignment (gpagen function in the geomorph package) on a coordinate dataset (argument coord.data) in 2-D or 3-D (argument n.dim) and record the resulting shape coordinates and centroid size. Here, the shape coordinates are projected onto tangent space because pairwise distances among corresponding datasets later in the analysis are based on Euclidean distances and most shape analyses in the biological literature are conducted in tangent space instead of Procrustes shape space.
2. Subsample the same three randomly selected landmarks from all specimens. The subsampling begins with a subset of three landmarks because a minimum of three landmarks is required to define shape. Then, generate shape and centroid size data with generalized Procrustes superimposition on the subsampled data.
3. Perform an Ordinary Procrustes Alignment (protest function in the vegan R package) on the subsampled data to minimize the pairwise distances between corresponding landmarks in the parent data through translation, rotation, and scaling of data in full shape space. As stated above, note that this procedure is different from the typical implementation of Procrustes alignment on coordinate data in 2-D or 3-D space. The function then records the sum of these squared distances as measure of fit (PSS) between the relative locations of data points in the parent and subsampled datasets. Equivalent procedure is conducted on centroid size data.
4. Sample one additional, randomly chosen landmark and repeat step 3 until the entire set of landmarks in the parent dataset is sampled. The completion of this step represents one iteration of subsampling.
5. Repeat steps 2–5 for specified number of iterations (iter argument).
6. Create sampling curves by plotting the trajectory of “fit” (1–PSS) against the number of landmarks sampled from each iteration for both shape and centroid size as gray lines. Then, plot the median fit value for each number of landmarks sampled on the same plot as a thick, dark line. Finally, output these sampling curves: LaSEC_SamplingCurve_Shape.pdf, LaSEC_SamplingCurve_Size.pdf.

### Data

To demonstrate its performance and utility, I performed LaSEC on published empirical datasets as well as simulated shape data with varying numbers of specimens, landmarks, and covariance structure. The four empirical datasets analyzed in this study include 2-D data of wasp wings with 19 landmarks and 249 specimens [24], 3-D cranial landmarks from an ontogenetic sampling of 10 extant species of crocodylians comprising 204 specimens and 78 discrete landmarks [25], 3-D landmark data from the condylar surface of femora in placental mammals that consist of 321 equally spaced surface semi-landmarks on 282 specimens [26], and 3-D craniofacial data of baboons which include 231 landmarks and semi-landmarks from 250 specimens from the NYCEP PRImate Morphometrics Online (PRIMO) database (http://primo.nycep.org). To examine the effect of specimen and landmark sampling on characterizations of shape variation, I simulated 2-D and 3-D coordinate data using the sim.coord function, also included in the LaMBDA R package. This function allows the user to generate coordinate data with specified number of specimens and landmarks from a normal distribution with a variance-covariance structure using the mvrnorm function in the MASS R package [27]. In this study, I conducted LaSEC on simulated 2-D and 3-D data with combinations of 10, 20, 40, and 80 specimens and landmarks and covariation values of 0.1 and 0.5 between pairs of coordinate variables.

## Results

**Fig 2:**
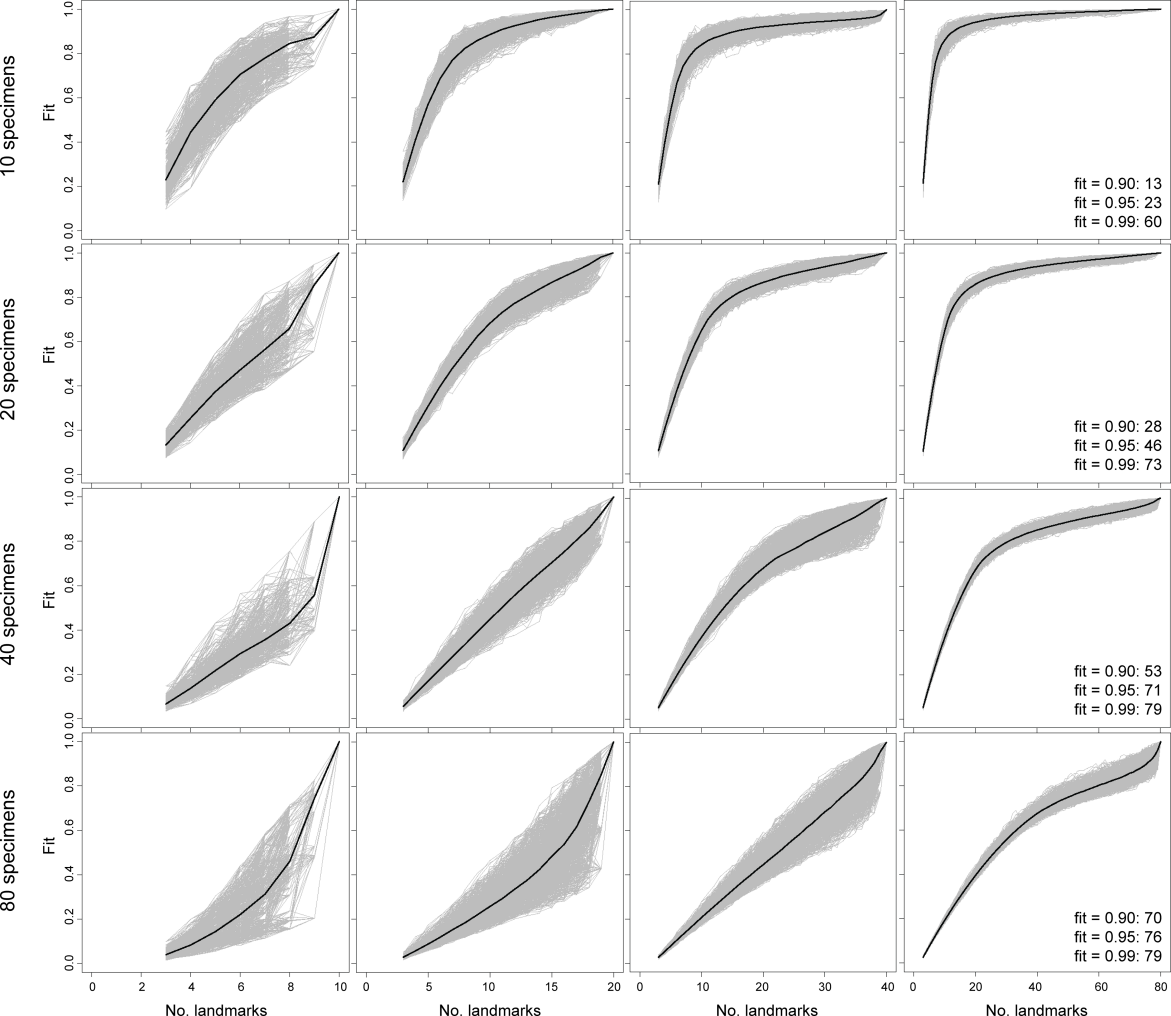
Sampling curves from performing LaSEC on a simulated 2-D dataset of simulated shapes with variable number of specimens and landmarks with covariance of 0.1. Each gray line indicates fit values from one iteration of subsampling. Thick, dark line denotes median fit value at each number of landmarks. Numbers within plots are the number of landmarks at median fit value of 0.90, 0.95, and 0.99.

**Fig 3:**
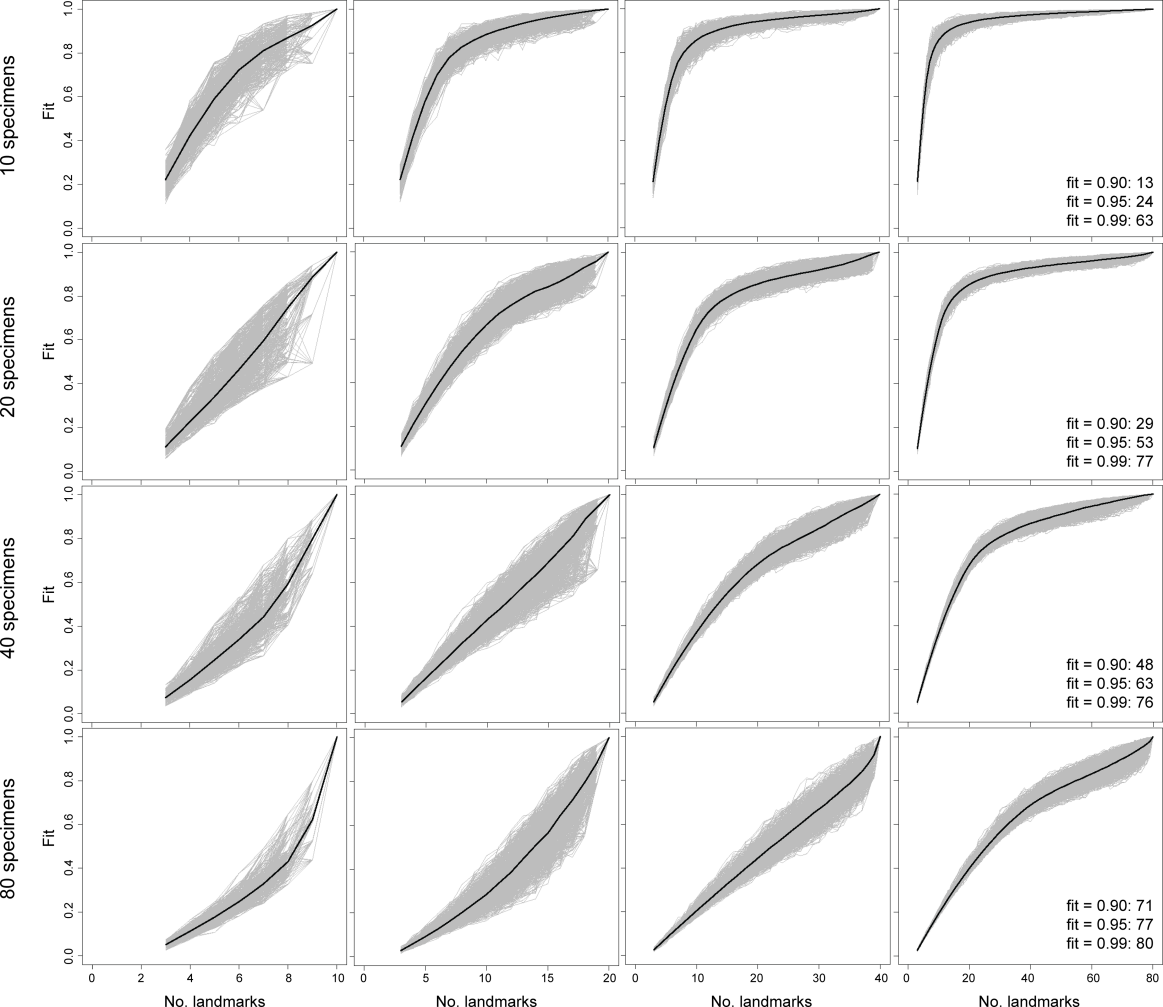
Sampling curves from performing LaSEC on a simulated 2-D dataset with variable number of specimens and landmarks with covariance of 0.5. Each gray line indicates fit values from one iteration of subsampling. Thick, dark line denotes median fit value at each number of landmarks. Numbers within plots are the number of landmarks at median fit value of 0.90, 0.95, and 0.99.

**Fig 4:**
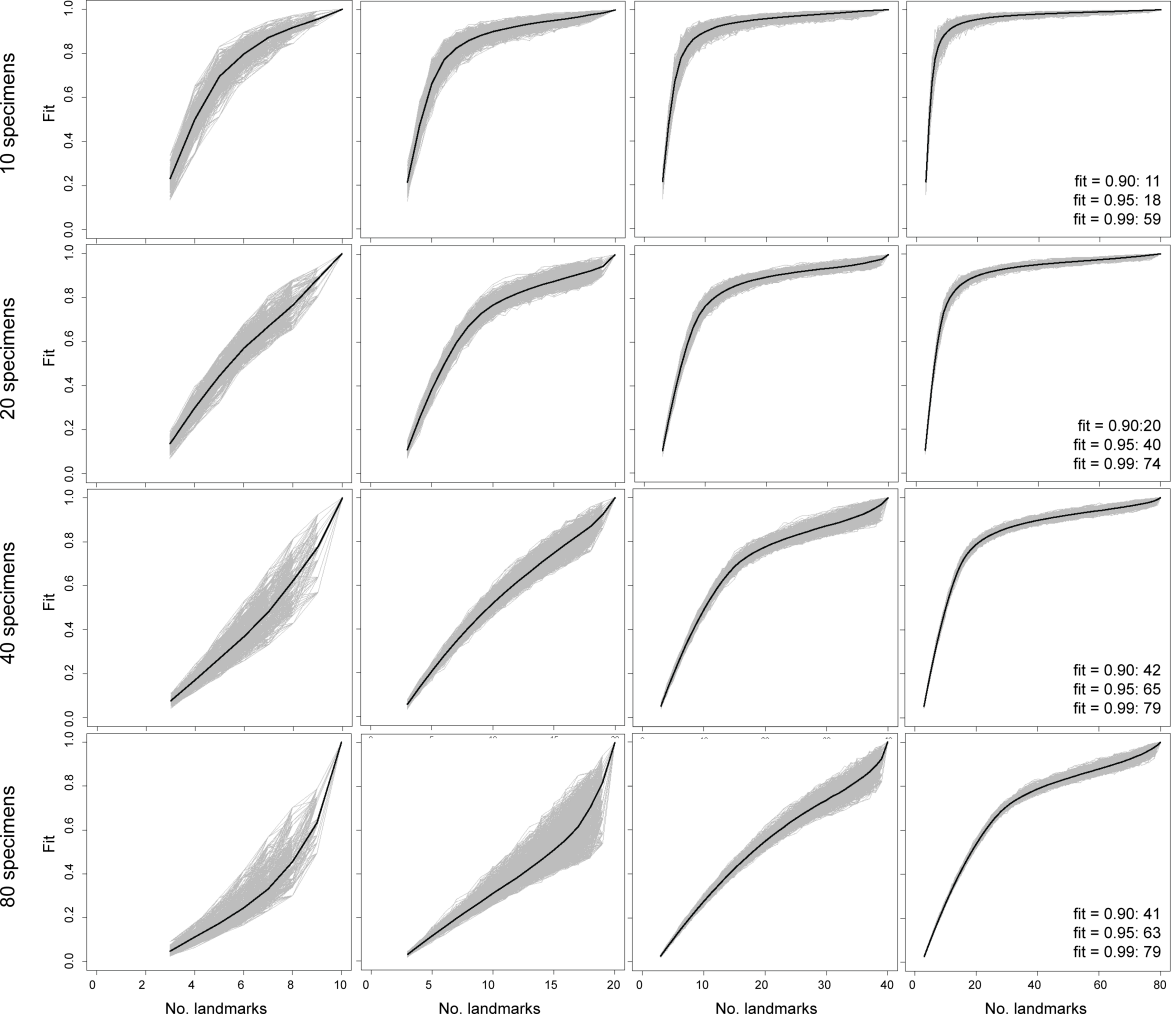
Sampling curves from performing LaSEC on a simulated 3-D dataset with variable number of specimens and landmarks with covariance of 0.1. Each gray line indicates fit values from one iteration of subsampling. Thick, dark line denotes median fit value at each number of landmarks. Numbers within plots are the number of landmarks at median fit value of 0.90, 0.95, and 0.99.

**Fig 5:**
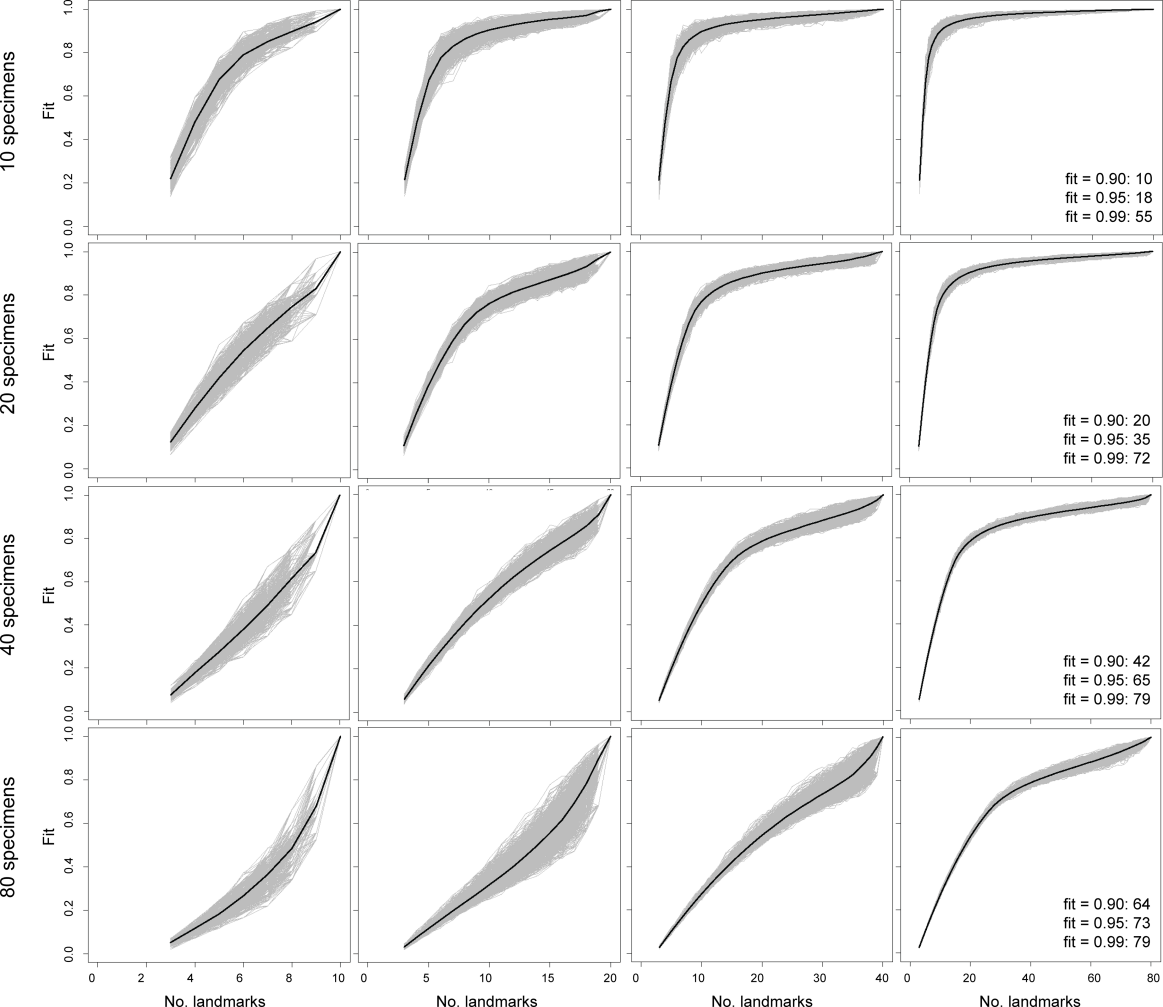
Sampling curves from performing LaSEC on a simulated 3-D dataset with variable number of specimens and landmarks with covariance of 0.5. Each gray line indicates fit values from one iteration of subsampling. Thick, dark line denotes median fit value at each number of landmarks. Numbers within plots are the number of landmarks at median fit value of 0.90, 0.95, and 0.99.

Comparisons of sampling curves from simulated data reveal clear indicators of robust shape characterization (Figs 2–5). First, and perhaps the most obvious, indicator is the presence of a plateau in fit values for datasets with greater numbers of landmarks than number of specimens. For simulated datasets with relatively poor landmark sampling (e.g., 80 specimens, 10 landmarks), the sampling curves do not show any decreases in slope as landmark sampling improves, implying a lack of stationarity in characterizing shape variation. Second, simulated data with relatively rich landmark sampling exhibit diminishing variance in fit values for a given number of landmarks as landmark sampling approaches that of the parent dataset. In contrast, datasets with poor landmark sampling show increasing variance in fit value. Taken together, the results from simulated data establish two indicators of stationarity in shape information: an extensive plateau in the sampling curve and diminishing variance in fit values. For simulated 2-D and 3-D data, these signs are observed when the number of landmarks exceeds specimen number.

Beyond these indicators of stationarity, performing LaSEC on simulated datasets elucidates the potential impact of covariation and physical dimensionality of landmarks (2-D or 3-D) in capturing shape variation. Given 2-D or 3-D data, the contour of the sampling curves is nearly identical between datasets with covariance values of 0.1 and 0.5. Likewise, the number of landmarks at median fit values between simulated datasets are fairly similar between the two covariance values (Figs 2–5: text in right-most plots). However, lower covariation noticeably increases the variance in fit values, particularly when landmark sampling is relatively poor. Comparisons between simulated 2-D and 3-D datasets suggest that 3-D landmarks are associated with lower variance in fit values and emergence of plateaus with relatively fewer landmarks than 2-D data. This observation is corroborated by fewer 3-D landmarks generally required to attain median fit values of 0.9 and 0.95. These results suggest that, given equivalent covariation structure, 3-D landmarks are more effective at capturing shape variation than 2-D landmarks because they are able to contain more information (i.e., additional z coordinates) that help distinguish the shapes of specimens.

**Fig 6:**
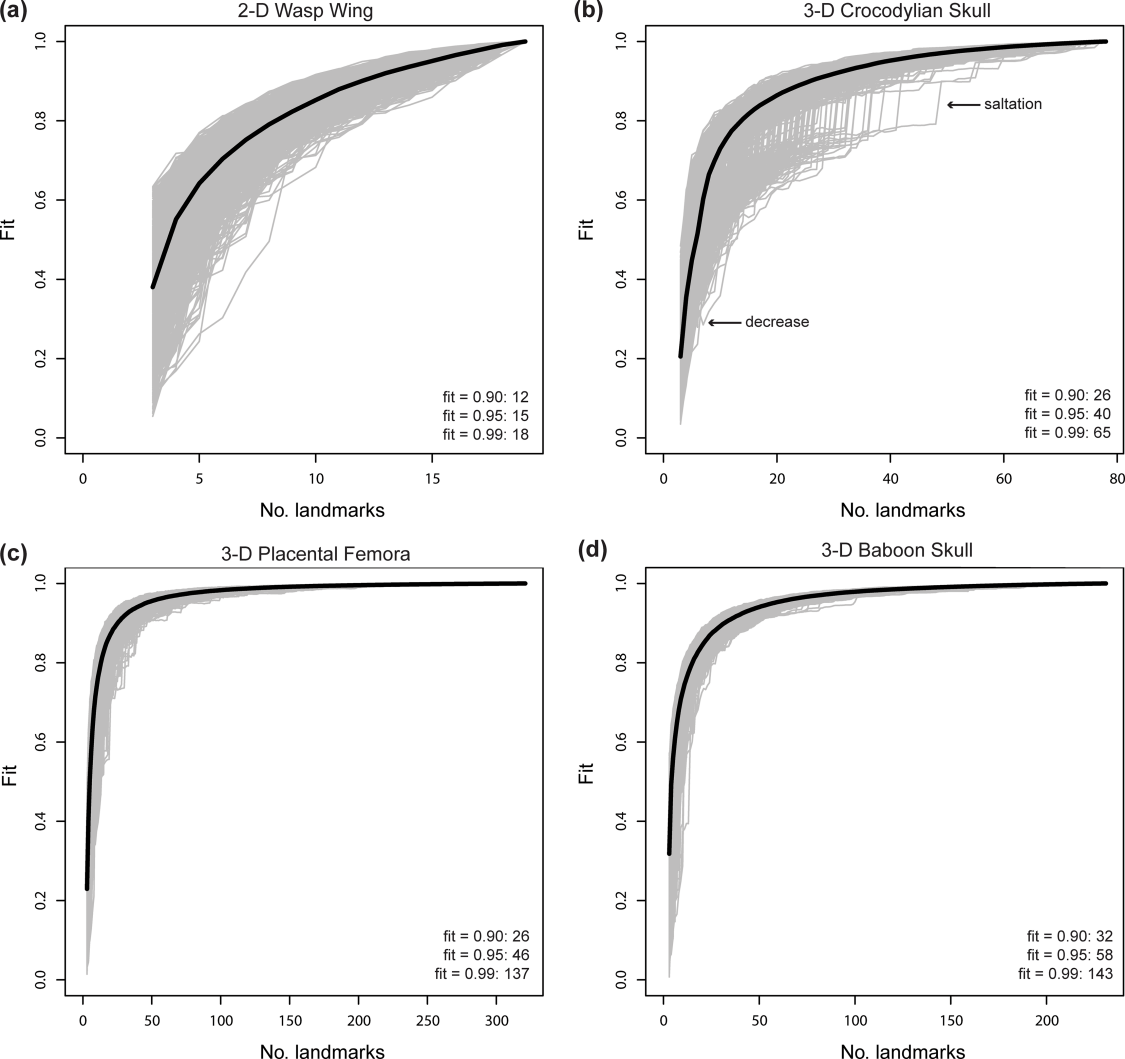
Sampling curves from performing LaSEC on empirical datasets with respect to characterizing shape variation. (a) 2-D wasp wing data [24]. (b) 3-D crocodylian skull data [25]. Arrows point to instances of saltations and decrease in fit value. (c) 3-D condylar surface semi-landmark data of placental mammal femora [26]. (d) 3-D baboon skull data. Each gray line indicates fit values from one iteration of subsampling. Thick, dark line denotes median fit value at each number of landmarks. Numbers within plots are the number of landmarks at median fit value of 0.90, 0.95, and 0.99.

The empirical datasets indicate varying levels of stationarity in the characterization of shape variation. The sampling curve from the wasp wing data (Fig 6A) resembles that of simulated data with 10 specimens and 10 landmarks. Despite the lack of a distinct plateau, the variance in fit values steadily decreases as the landmark sampling approaches the parent dataset. This result suggests that the convergence to the parent dataset is genuine although confirming the authenticity of the asymptotic trajectory requires sampling additional landmarks to extend the sampling curve. In this dataset, the median fit values of 0.90 and 0.95 require 12 and 15 landmarks (63.1, 78.9%), respectively, of the 19 total landmarks.

Relative to the wasp wing data, the crocodylian data exhibit a more robust plateau (Fig 6B) and proportionately fewer landmarks (33.3 and 51.3% of 78 landmarks respectively) to reach median fit values of 0.90 and 0.95. Notably, the addition of certain landmarks shows marked improvement in fit values (Fig 6B: top arrow). These saltations in fit signify that some landmarks are critical for capturing shape information (e.g., landmarks at the edges of structures that anchor the alignment and global signal in shape variation).

Finally, the placental femur and baboon skull data generate sampling curves that resemble those from simulated data with 10 specimens and 80 landmarks (Fig 6C and 6D). This observation implies that these datasets have robustly characterized shape variation. For these datasets, merely 14.3% and 25.1% of landmarks are needed to capture equivalent fidelity in shape information (fit ≥ 0.95), respectively. As such, a substantial portion of the landmarks and semi-landmarks from these datasets could be removed while retaining the fidelity in characterizing shape variation.

Based on both empirical and simulated shape data, the sampling curves (Figs 2–6) show that landmark data are statistically consistent in characterizing shape variation. Although sampling one additional landmark may, in some cases, reduce the fit of a subsampled data set to the parent data (Fig 6B: bottom arrow), the median values for each subset of landmarks (Figs 2–6: dark lines) consistently converge to the pattern of shape variation of the parent dataset. Therefore, landmark-based geometric morphometric data generally converge to the shape distribution of the parent dataset as more landmarks are sampled. In contrast, the evidence of statistical efficiency of landmark-based data is mixed. For empirical datasets, the variance in fit values diminishes as additional landmarks are sampled (Fig 6), whereas sampling curves from simulated shape data (Figs 2–5) show increasing variance in fit initially, followed by decreasing variance after an inflection point (i.e., location of highest curvature).

**Fig 7:**
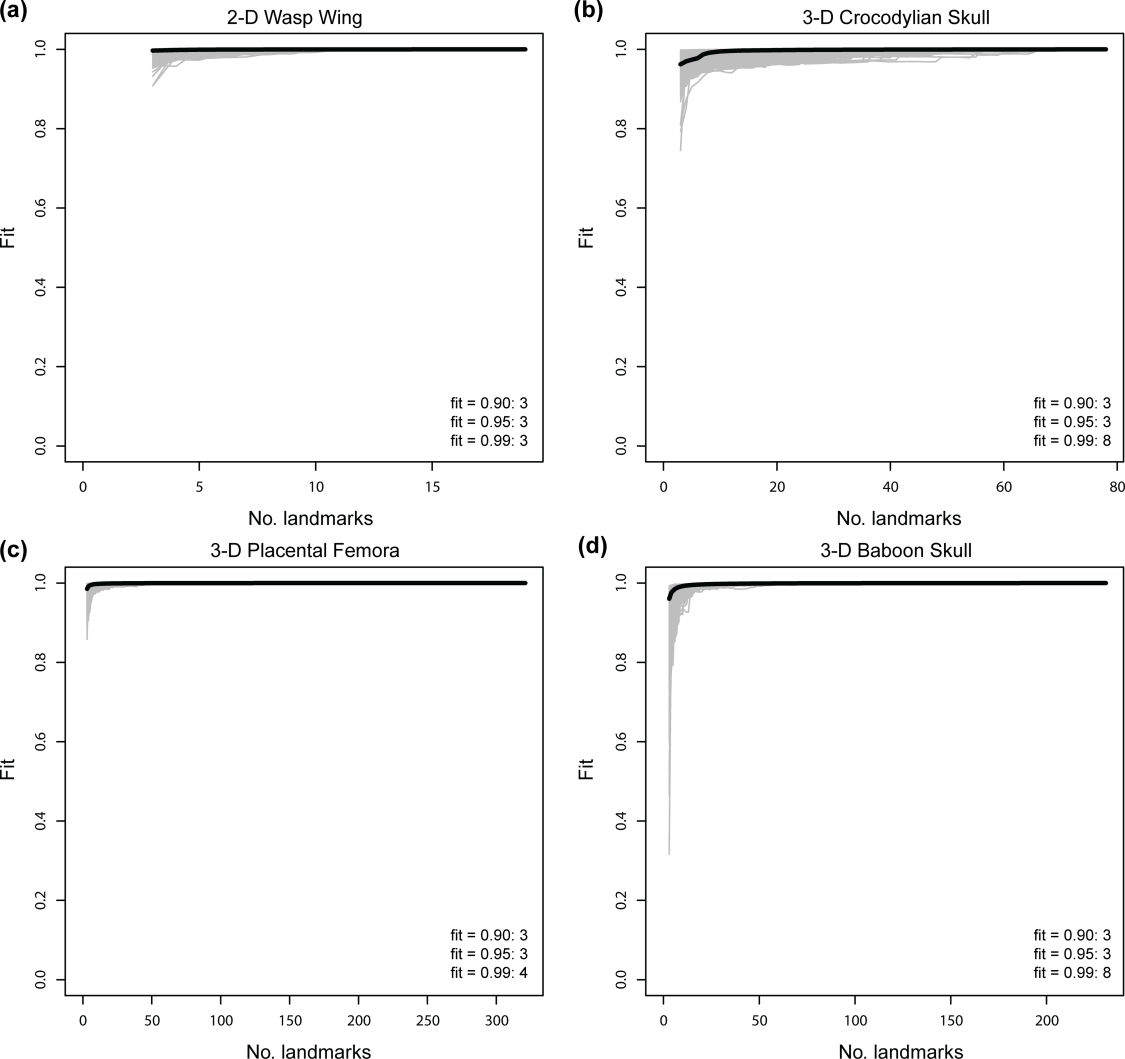
Sampling curves for subsampled empirical data with respect to characterizing centroid size variation. (a) 2-D was wing data [24]. (b) 3-D crocodylian skull data [25]. (c) 3-D condylar surface semi-landmark data of placental mammal femora [26]. (d) 3-D baboon skull data. Each gray line indicates fit values from one iteration of subsampling. Thick, dark line denotes median fit value at each number of landmarks. Numbers within plots are the number of landmarks at median fit value of 0.90, 0.95, and 0.99.

Sampling curves for centroid size illustrate that very few landmarks are necessary to accurately capture size variation among specimens (Fig 7). In all four data sets, high fidelity in size information (fit ≥ 0.95) requires only three landmarks (i.e., minimum number of landmarks required to define a shape). No more than eight landmarks are needed to reach a median fit value of 0.99 for any empirical data set examined here.

## Discussion

### Properties of landmark data

Running LaSEC on multiple types of data demonstrates important properties of landmark-based GM data. The effect of inter-landmark covariance structure on shape characterization is unclear based on simulated data analyzed in this study. As anticipated, however, the results suggest that fewer 3-D than 2-D landmarks are generally required to attain equivalent fidelity in shape characterization. In addition, both simulated and empirical data show that landmark data are statistically consistent for capturing shape and size variation, where incorporating additional landmarks will steadily improve the characterization of shape variation, at least as measured by PSS. Whether landmark data are statistically efficient is less clear. For the empirical datasets used in this study, the variance in fit value diminishes consistently with larger landmark sampling, implying that landmark data are statistically efficient (Fig 6). The sampling curves of simulated datasets, in contrast, are more nuanced, showing increasing variance in fit values up to certain subsampling of landmarks, followed by a decreasing trend once the landmark sampling reaches a plateau in the curve (Figs 2–5). These conflicting outcomes suggest that theoretically, landmark data are not necessarily statistically efficient, but they are likely to be efficient for characterizing shape variation in biological systems.

Similar discrepancy exists between simulated and empirical data regarding the number of landmarks required for stationarity in shape information. Simulated 2-D and 3-D data suggest that stationarity in shape information requires the number of landmarks to exceed the number of specimens (Figs 2–5). With the exception of simulated data with 80 specimens, landmark sampling reaches a median fit value of 0.90 only when the number of landmarks equals or exceeds the specimen count. Such data could dramatically reduce the power of parametric statistical tests or prohibit certain procedures, although recent methodological advancements have mitigated this issue for some analyses [10,28,29]. The empirical data selected for this study, however, indicate that equivalent fidelity in shape information is achieved with fewer landmarks than specimens (Fig 6). Again, these discordant results are likely due to fundamental differences in the structure of shape variation between simulated and empirical datasets. Compared to coordinates sampled from a normal distribution under a single variance-covariance scheme, shape variation in biological systems is expected to be far more structured due to myriad factors, such as physical, developmental, and functional constraints, that differentially act on taxonomic groups and anatomical structures. Hence, proportionately far fewer landmarks are expected to be necessary for robustly capturing the shape variation of biological specimens than with simulated shapes.

Nevertheless, landmark data will each contain a unique structure in shape variation. For instance, denser landmark sampling is generally expected for capturing morphological variation within a population than across major clades. While analyzing simulated data helps identify clear signs of robust shape characterization, the idiosyncrasies of individual datasets prevent a formulation of unambiguous principles regarding adequate number of landmarks that are extensible across all empirical datasets. As such, the quality of landmark data need to be assessed for individual datasets.

In addition to shape characterization, this study also clearly shows that landmarks capture size variation very effectively, requiring no more than eight landmarks in all empirical data sets examined in this study (Fig 7). This result corroborates a previous study indicating that small intraspecific sampling provides accurate estimation of mean centroid size, whereas more than 20 specimens are needed to estimate the mean shape of species [5]. Related studies have also shown that one-sided data are able to characterize size variation as effectively as bilaterally sampled data [6,7]. This efficacy of landmarks in characterizing size variation is expected because size is a univariate trait that correlates strongly with linear distances measured on corresponding specimens. Because inter-landmark distances are inherent in landmark data, very few landmarks are typically needed for robust characterization of size variation.

### A guide for using LaSEC

LaSEC is an exploratory tool for assessing whether a given landmark sampling robustly characterizes the shape variation of specimens under study. Its output provides three related but valuable pieces of information: (1) stationarity in shape and size information; (2) evidence of over- or under-sampling of landmarks; and (3) minimum number of landmarks needed to retain equivalent shape information. The function LaSEC is included in the LaMBDA R package (www.github.com/akiopteryx/lambda), and performed by running lasec(coord.data, n.dim, iter, show.progress), where coord.data is the coordinate data in a 2-D matrix format (rows of specimens and columns of coordinate variables), n.dim denotes the physical dimensionality of the data (2 or 3 for 2-D and 3-D data respectively), iter specifies the number of rounds of subsampling (default is 1,000), and show.progress is the option to display a progress bar during the analysis. Although this study focuses on biological systems, LaSEC can be run on any landmark-based coordinate data.

After the completion of subsampling rounds, LaSEC produces sampling curves that visualize the degree of stationarity in characterizing shape variation among specimens. Distinct signs of stationarity include the presence of a plateau and diminishing variance in fit value. Absence of these features (e.g., Fig 6A) implies that the given landmark scheme is unreliable with respect to capturing shape variation. Consequently, re-evaluation of data with additional landmarks is necessary to ensure that accurate shape information is being collected. The adequate number of additional landmarks will depend on the intended specimen sampling, the morphological features under study, as well as the locations and definitions of individual landmarks. In some cases, sampling additional landmarks may be difficult. If discrete anatomical landmarks have been exhausted, one can consider employing curve or surface semi-landmarks for denser characterization of shape [9,14]. Other cases where additional landmarks would exclude damaged, but critical, specimens may require imputation and retrodeformation techniques to estimate missing or deformed landmarks [30–32].

Alternatively, if the sampling curve exhibits indicators of robust characterization of shape variation, then the user could perform subsequent analyses with the current landmark scheme or remove a proportion of landmarks while maintaining equivalent shape information. For the latter case, the sampling curve provides a visual guide to how many landmarks can be removed confidently without jeopardizing the characterization of shape variation. A list of median fit values (output median.fit) offers a more quantitative approach, where the user could remove a certain number of landmarks down to a specific fit value (e.g., fit > 0.95). In the crocodylian dataset, for example, a median fit value of 0.95 is maintained even with the removal of 28 of the 78 landmarks (Fig 6B). The issue of precisely which landmarks to remove is discussed below but ultimately decided by the investigator. Once landmark sampling is appropriately reduced, performing LaSEC on a new dataset is unnecessary. Even if the sampling curve of the new dataset lacks signs of stationarity, prior analysis has already demonstrated that the shape variation characterized by the new landmark scheme is consistent with that of the parent dataset. By providing qualitative and quantitative justifications for removing landmarks, LaSEC has the potential to reduce the work load of future data collection, maintain power in downstream analyses, and incorporate specimens with missing landmarks.

Furthermore, conducting LaSEC on previously published landmark data helps the user evaluate the reliability of the results and conclusions. For instance, the output from the crocodylian dataset suggests that approximately 40 3-D landmarks are needed to accurately characterize the shape variation in extant crocodylians (Fig 6B). Yet, Foth and colleagues collected only 20 2-D landmarks from the skulls of the extant crocodylian *Melanosuchus niger* [33]. Although their smaller landmark sampling may capture the intraspecific shape differences within *M*. *niger*, 40 3-D landmarks are needed to characterize the more pronounced interspecific shape variation. Therefore, additional landmarks should be collected to be more certain that the finer intraspecific shape variation in *M*. *niger* is being accurately characterized.

Given the crucial information it provides concerning data quality, I recommend performing LaSEC in the initial phases of data collection, where the user has sample landmarks from specimens that collectively exhibit the breadth of morphological variation intended for the study. Following this recommendation allows the investigator to determine whether greater or fewer numbers of landmarks will be required for generating reliable results. If landmark sampling is found to be inadequate, landmark sampling can be augmented to prevent further collection of undersampled, potentially inaccurate, data. Conversely, if LaSEC suggests stationarity in characterization of shape variation, then a strategic reduction of landmarks could maximize sample size through faster digitization of specimens and accommodation of specimens with missing landmarks.

### Limitations of LaSEC

Although LaSEC provides valuable information on the quality of landmark data, it has few notable limitations, at least in the current version. First, the analysis does not determine a precise number or proportion of landmarks that could be removed from the dataset. While the number of landmarks at median fit values of 0.90, 0.95, and 0.99 could inform the number of landmarks to remove, these cutoff points are arbitrary. This situation is akin to evaluating the amount of burn-in to remove in a Bayesian analysis, where the user decides how much data to remove based on the contour of the plot.

Second, the analysis does not identify which landmarks could be removed with minimal consequences to data quality. Due to the naïve selection of landmarks in LaSEC, some subsamplings of landmarks are anticipated to be impractical and suboptimal (e.g., sampling landmarks only from a restricted region of a structure). Hence, an informed removal of landmarks by an investigator will likely result in greater congruence with the parent dataset than the median fit values given by LaSEC. In practice, the user should confirm a high congruence (e.g., fit ≥ 0.95) between an informed subsampled data to the parent data set using the protest function in the vegan R package [19]. To illustrate, I removed 35 landmarks on the left side of the skull from the crocodylian dataset to compare the parent dataset with landmark data comprising median and right landmarks. The resulting fit value was 0.999 compared to median fit value of 0.959 with the same number of landmarks (43 of 78 landmarks), indicating that an informed subsampling will be superior to subsets of randomly selected landmarks with respect to shape characterization.

Finally, LaSEC does not consider sliding semi-landmark alignments [9,15] due to challenges associated with tracking adjacent semi-landmarks during the subsampling procedure. Moreover, implementation of sliding semi-landmarks may be insensible and ultimately cause difficulty in the interpretation of results because the direction of sliding will fluctuate dramatically based on random and often sparse subsampling of neighboring semi-landmarks. In this study, empirical datasets with semi-landmarks were analyzed to examine results from data with dense landmark sampling.

## Conclusions

Despite these current limitations, LaSEC, included in the new LaMBDA R package, equips investigators with a tool to systematically evaluate the quality and properties of landmark-based GM data. Simulated datasets clearly establish two indicators of reliable landmark data: the presence of a plateau in the resulting sampling curve and diminishing variance in fit values as landmark sampling approaches the full set of landmarks. Both empirical and simulated data demonstrate statistical consistency in landmark data for characterizing shape (and size) variation. While landmark data are not necessarily statistically efficient in this task, empirical datasets suggest that they are efficient at characterizing variation in biological shape. As landmark-based GM data continue to accumulate at a rapid pace, LaSEC places the focus on the equally important task of evaluating and refining these data—one that is vital to achieving accurate understanding of morphological variation.

## Supporting information

S1 Fig Sampling curve of crocodylian skull data based on correlation coefficients from two-block partial least squares analysis. Note high correlation between subsampled and parent datasets despite low visual correspondence in morphospace (Fig 1).
(TIF)

S1 Data and Code ZIP-archived directory containing all computer programs. Note that the code will not be updated after publication. The most recent version is available via GitHub: www.github.com/akiopteryx/lambda.
(ZIP)
