# Supplementary figures and images for: How many landmarks are enough to characterize shape and size variation?
Source: PLoS One. 2018 Jun 4;13(6):e0198341. doi: 10.1371/journal.pone.0198341 (PMC5986137; doi:10.1371/journal.pone.0198341)

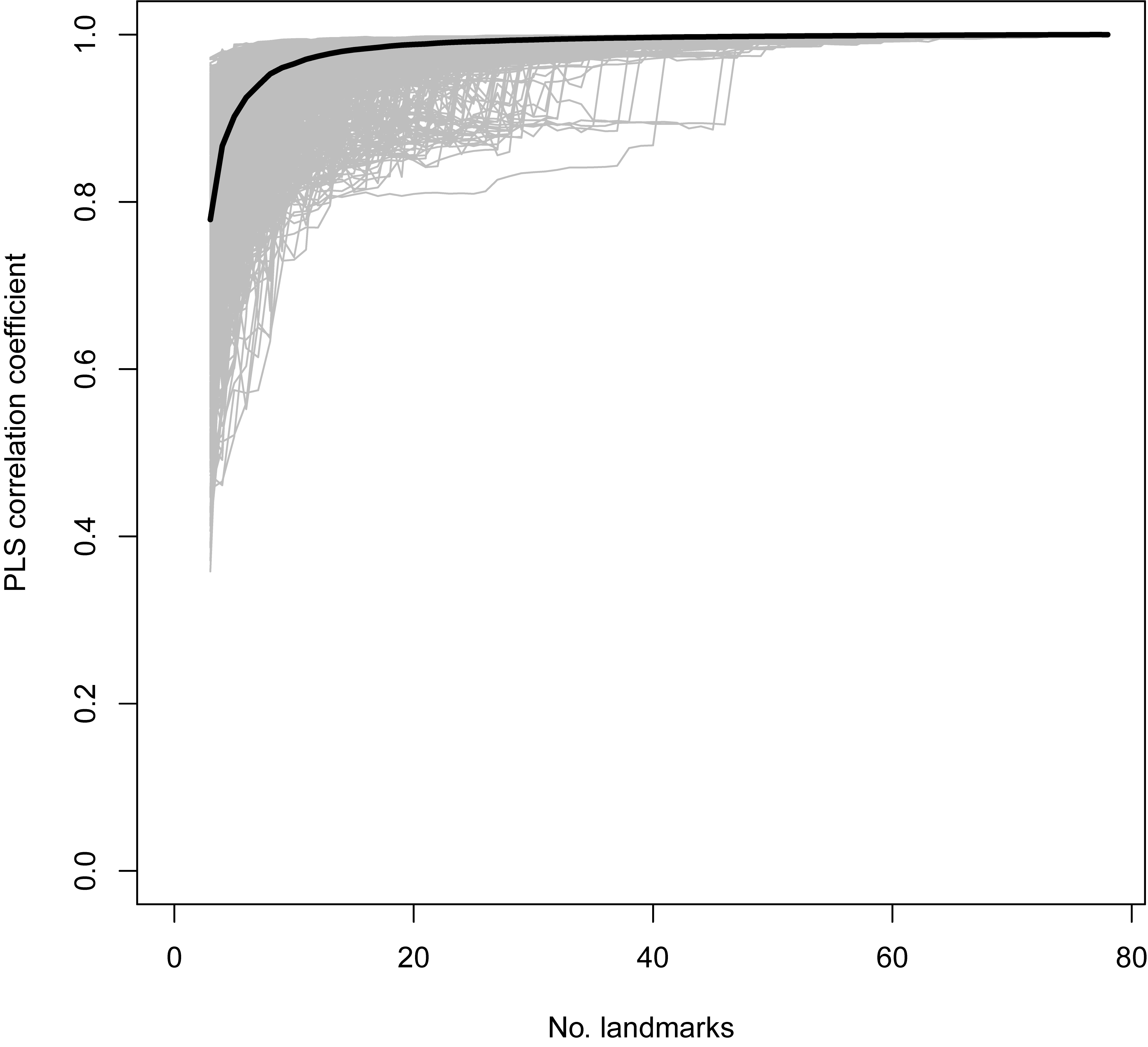

Supplement: S1 Fig — Note high correlation between subsampled and parent datasets despite low visual correspondence in morphospace (Fig 1). (TIF) [file pone.0198341.s001.tif]
